# Causal relationship from heart failure to kidney function and CKD: A bidirectional two-sample mendelian randomization study

**DOI:** 10.1371/journal.pone.0295532

**Published:** 2023-12-11

**Authors:** Junyu Zhang, Zhixi Hu, Yuquan Tan, Jiahao Ye

**Affiliations:** 1 Institute of Traditional Chinese Medicine Diagnostics, Hunan University of Chinese Medicine, Changsha, Hunan, China; 2 Hunan Provincial Key Laboratory of Diagnostic Research in Chinese Medicine, Hunan University of Chinese Medicine, Changsha, Hunan, China; Tehran University of Medical Sciences, ISLAMIC REPUBLIC OF IRAN

## Abstract

**Background:**

Heart Failure (HF) is a widespread condition that affects millions of people, and it is caused by issues with the heart and blood vessels. Even though we know hypertension, coronary artery disease, obesity, diabetes, and genetics can increase the risk of HF and Chronic Kidney Disease (CKD), the exact cause of these conditions remains a mystery. To bridge this gap, we adopted Mendelian Randomization (MR), which relies on genetic variants as proxies.

**Methods:**

We used data from European populations for our Bidirectional Two-Sample MR Study, which included 930,014 controls and 47,309 cases of HF from the HERMES consortium, as well as 736,396 controls and 51,256 cases of CKD. We also employed several MR variations, including MR-Egger, Inverse Variance Weighted (IVW), and Weighted Median Estimator (WME), to guarantee the results were accurate and comprehensive.).

**Results:**

In this study, the MR analysis found that individuals with a genetic predisposition for HF have an elevated risk of CKD. Our study revealed a significant association between the genetic prediction of HF and the risk of CKD, as evidenced by the IVW method [with an odds ratio (OR) of 1.12 (95% CI, 1.03–1.21), p = 0.009] and the WME [with an OR of 1.14 (95% CI, 1.03–1.26), p = 0.008]. This causal relationship remained robust even after conducting MR analysis while adjusting for the effects of diabetes and hypertension, yielding ORs of 1.13 (IVW:95% CI, 1.03–1.23), 1.12 (MR-Egger: 95% CI, 0.85–1.48), and 1.15 (WME:95% CI, 1.04–1.27) (p = 0.008). However, in the reverse analysis aiming to explore CKD and renal function as exposures and HF as the outcome, we did not observe a statistically significant causal link between CKD and HF.

**Conclusion:**

Our study demonstrates the significance of HF in CKD progression, thus having meaningful implications for treatment and the potential for discovering new therapies. To better understand the relationship between HF and CKD, we need to conduct research in a variety of populations.

## 1 Introduction

Heart Failure (HF) is an intricate syndrome of many heart conditions stemming from either structural or functional issues with the heart. It’s a global problem, impacting around 40 million individuals [1]. High blood pressure, blocked arteries in the heart, diabetes, being overweight, smoking and family history are all common causes of HF [2]. When diagnosing HF, it is important to consider symptoms, the physician’s diagnosis, and any objective characteristics of the disease [3]. Treating HF mainly involves making lifestyle changes and taking antihypertensive medications, such as angiotensin II receptor blockers (ARBs), ACE inhibitors, and β-blockers [4]. Meanwhile, studies have demonstrated that the outlook for HF is typically worse, and Chronic Kidney Disease (CKD) is a key indicator for the prognosis of HF [5].

CKD is a progressive illness identified by structural damage to the kidneys and decreased kidney function [6]. It is estimated that over 20 million Americans are affected by CKD, primarily caused by diabetes and hypertension [7]. Glomerular Filtration Rate (GFR) is often used to detect CKD, and serum creatinine is used to estimate GFR [8,9]. Treatment for CKD is analogous to that of HF, including implementing lifestyle modifications and beginning medication use promptly to reduce the effects of the underlying causes, like diuretics to lower blood pressure or insulin to reduce the burden on the liver and kidneys [7].

According to the findings of a multi-center study, approximately one-fifth to two-fifths of those afflicted with acute HF have renal dysfunction, while about four-fifths to six-tenths of those with chronic HF have CKD [10]. Research has revealed a correlation between HF and CKD, yet their causal relationship has not yet been examined in detail [11,12]. Therefore, it is still uncertain whether HF is a contributory factor in the onset of CKD.

To estimate the effect of exposure on outcomes, mendelian randomization (MR) is a method of instrumental variable analysis that takes advantage of genetic variants closely linked to the exposure as instrumental variables (IVs) [13]. Genetic factors and single nucleotide polymorphisms (SNPs) as exposure evidence can circumvent reverse causality and other mistakes compared to traditional analysis techniques [14]. In order to determine if there is a cause-and-effect between HF and CKD, we need first to identify the underlying causes of CKD. This study examined the potential correlation between Urinary Albumin Creatinine Ratio (UACR) and Epidermal Growth Factor Receptor (EGFR) and CKD. Through MR analysis, we aimed to determine if the effect of HF on CKD is causal.

## 2 Materials and methods

### 2.1 Data source

**Fig 1:**
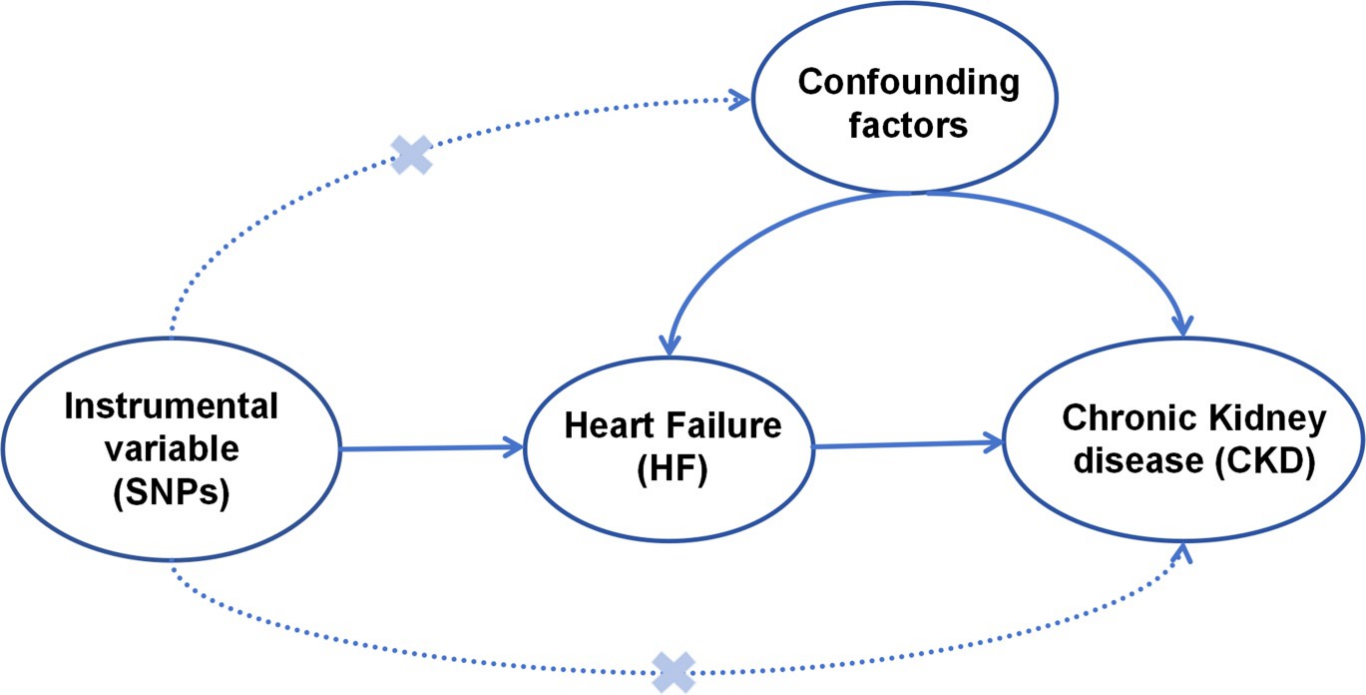
Plot of key assumptions for MR analysis.

We investigated the association between HF and CKD using a bidirectional two-sample MR technique. GWAS enabled us to compare the SNP loci spotted in the patient’s genome, bypassing the need to guess at the causative genes, as in a candidate gene approach [15]. Further details were listed in Fig 1.

A meta-analysis of 26 studies, including 930,014 controls and 47,309 HF cases, conducted by the Heart Failure Molecular Epidemiology for Therapeutic Targets (HERMES) (https://www.hermesconsortium.org/) consortium revealed 12 independent variants linked to HF in 11 genomic regions [16].

The CKDGen Consortium (http://ckdgen.imbi.uni-freiburg.de/) obtained pooled CKD and renal function data from two large-scale GWAS meta-analyses[17,18]. Specifically, the UACR-related data from Teumer et al. [17] was selected, which included 51861 cases and 297,093 control data (No UK Biobank database or research resources). Additionally, eGFR, BUN, and CKD data from Wuttke et al. [18] were included, with 41,395 cases and 439,303 controls. GWAS analysis was only conducted on people of European descent to reduce potential population stratification bias.

Ethical approval is unnecessary for this project since all the scores are available to the public.

### 2.2 Genetic variants selection criteria

The validity of an IV estimator was determined by its ability to fulfill three criteria: (1) a significant relationship between the instrumental variables and HF, (2) independence of the instrumental variables from all confounding factors of the HF-CKD association, and (3) the ability of the instrumental variables to only affect outcomes if they were related to HF [19]. For MR research to be valid, the three assumptions outlined must be satisfied. If the desired outcome was not achieved, it could be due to horizontal pleiotropy, linkage disequilibrium (LD), weak instrument, or population stratification.

A "weak instrument" is when the genetic variants are not significantly associated with the exposure factors. A distance-based metric was used to analyze a list of SNPs with a p-value of less than 5×10^−6^ to identify HF-related SNPs. The IVs thresholds of CKD and kidney function were set to *p*<5×10^−8^. The F-statistic in the regression model was employed to reduce the bias created by weak instrumental variables. As a rule of thumb, if the F-statistic of the instrumental variable was over 10, the weak instrument was disregarded [20].

A pleiotropic effect of a genetic variant could occur when other, unmeasured, confounding effects were linked to the outcomes, violating the assumption of exclusivity and independence. To counterbalance the pleiotropic effects of instrumental variables, Inverse Variance Weighting (IVW) [21], MR-Egger [22], and Weighted Median (WM) [23] were employed to create regression models that connected gene exposure and gene outcome, and thereby test and rectify any potential bias.

LD is a phenomenon in which genetic variants located close to each other are usually inherited together [24]. To minimize the LD effect and guarantee the independence of IVs, the genetic distance was at least 10000 kb, and the LD of IVs was no greater than 0.001. SNPs related to exposure factors were taken from the GWAS dataset of outcome variables, recording information such as IVs, allele effect size (β), effector alleles, standard error (se), and p-value.

To prevent population stratification, we only included populations from Europe, thereby avoiding any population deviation caused by variations in the frequency of genetic variants across populations with different genetic backgrounds.

### 2.3 Mendelian randomization analysis

Researchers have employed a range of approaches, such as inverse variance weighted (IVW), Weighted median (WM), MR-egger regression, and MR-PRESSO, to explore the potential causal connection between HF and CKD. IVW, a standard approach, was used as the primary calculation, combining data from several instruments to produce the ratio. Assuming all SNPs were valid, IVW offered an unbiased estimate of the results without horizontal pleiotropy. However, heterogeneity or off-target genetic effects could lead to the inaccuracy of IVW.

We tested various models and assumptions of horizontal pleiotropy to ensure our estimates were accurate and consistent. No correlation between horizontal pleiotropic effects and SNP exposure was observed in the MR-Egger analysis, which mainly reflects the dose relationship between instrumental variables and results, accounting for some pleiotropy [25]. To reduce the risk of class I errors and accept some genetic variations, the weighted median method was used, wherein the weight of each SNP in the population estimate was determined by the accuracy of its ratio estimate [26]. MR-PRESSO eliminated SNP outliers (with a *p*-value of less than 0.05) which could introduce bias [27]. Even if some instrumental variables did not meet the criteria for causal inference, the weighted model approach was still an option as long as most instrumental variables had comparable causal estimates. If the results of these methods differ, IVW should be given priority as the primary outcome.

### 2.4 Pleiotropy and sensitivity analysis

MR is a reliable and effective statistical method for establishing causal relationships; however, factors such as imbalance, pleiotropy, and epigenetic influences can interfere with the principles of instrumental variables. To measure heterogeneity, we used the MR-Egger intercept and Cochran’s Q tests. Using MR-Egger regression is a great way to calculate the average pleiotropy effect; a regression equation with an intercept of 0 indicates the presence of pleiotropy [28]. MR-PRESSO was adopted to detect and resolve any anomalies in the IVW linear regression [29]. To evaluate the impact of individual SNPs, we performed a leave-one-out sensitivity analysis. The researchers used Phenoscanner [30] (http://www.phenoscanner.medschl.cam.ac.uk/) to examine the confounder candidates (including body mass index, hypertension, and diabetes) associated with the instrumental variables to consider their potential influence.

### 2.5 Statistical analysis

The R statistical program was used to analyze the data with Two Sample MR (version 0.5.6). If the p-value was less than 0.05, it was considered statistically significant.

## 3 Results

### 3.1 Effect of HF on CKD

**Fig 2:**
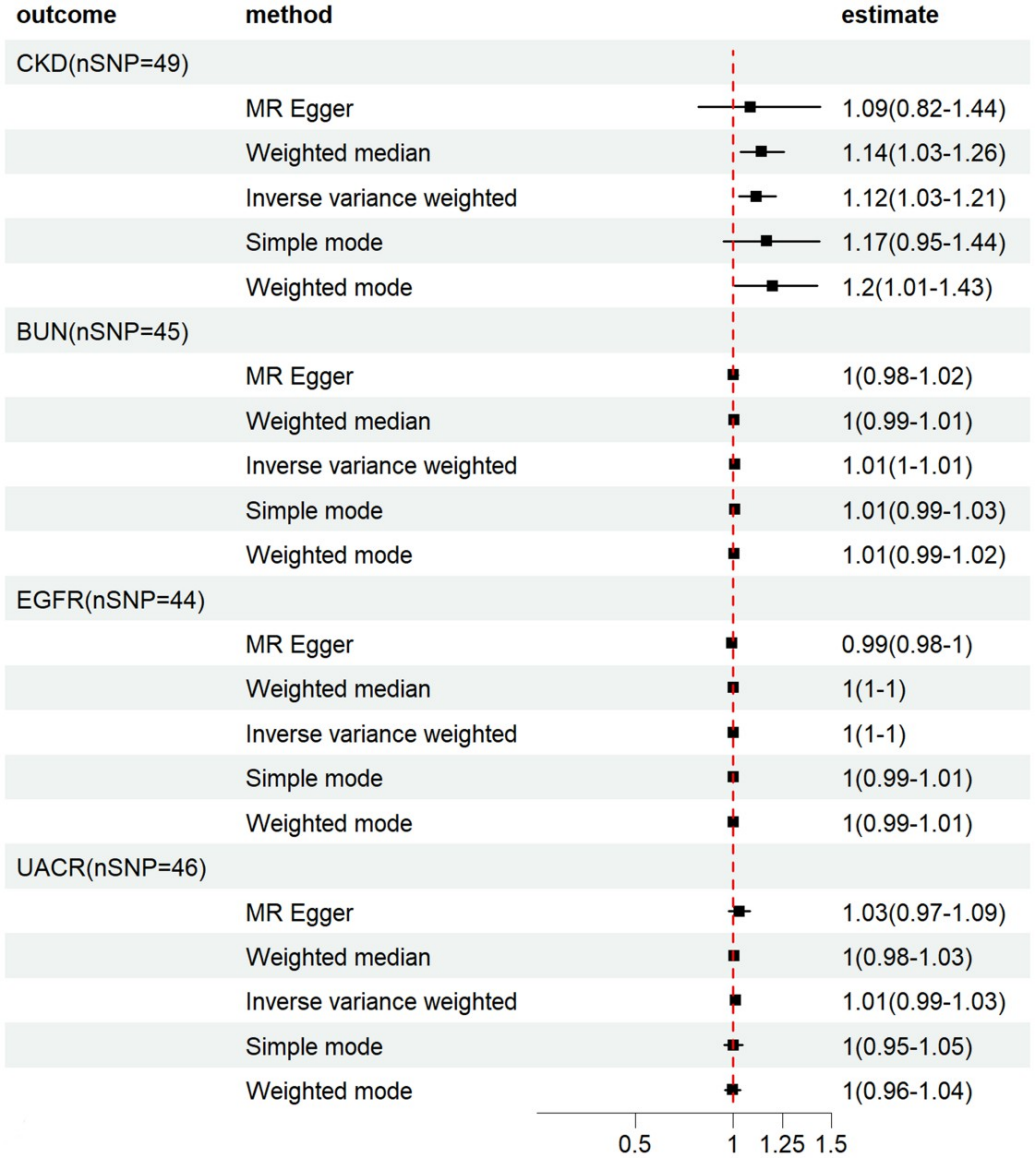
Forest plot of HF effects on CKD.

**Fig 3:**
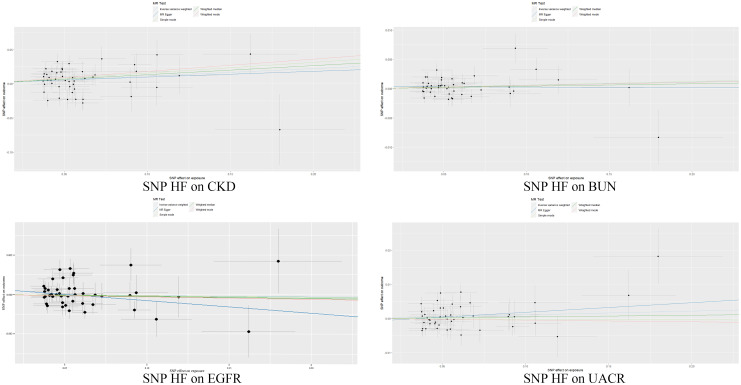
Map of intercept. The slope of each line corresponds to the estimated MR effect in different models.

Our study found that genetic prediction of HF had a significant correlation with the risk of CKD, as indicated by the IVW method [odds ratio (OR), 1.12 (95% CI, 1.03–1.21), *p* = 0.009]. MR-Egger [OR, 1.09 (95%CI, 0.82–1.44), *p* = 0.56] and WME[OR, 1.14 (95%CI, 1.03–1.26), *p* = 0.008] also showed similar results, with the *p*-value of MR-Egger method greater than 0.05. Furthermore, we found no causal relationship between HF-BUN [IVW: OR, 1.01(95% CI, 0.99–1.01), *p* = 0.13], HF-EGFR [IVW: OR, 1.00(95% CI, 1.00–1.00), *p* = 0.74] and HF-UACR [IVW: OR, 1.01(95% CI, 0.99–1.03), *p* = 0.20], which further supports the significance of our study(S10 Table). More details can be found in Figs 2 and 3.

**Fig 4:**
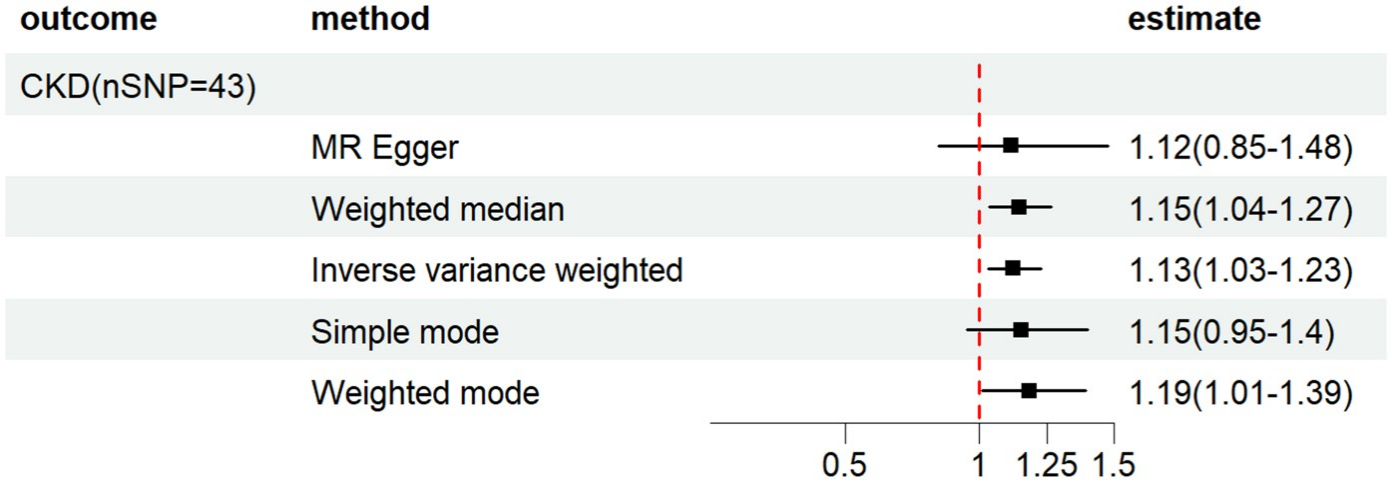
Forest plot of HF effects on CKD (Hypertension and Diabetes mellitus were excluded).

Next, a two-sample MR analysis was carried out to explore the connection between HF and CKD. To ensure that confounding factors, such as hypertension and diabetes, did not affect the results, we excluded SNPs associated with these conditions. Our second MR analysis showed that HF was significantly correlated with CKD risk, with OR of 1.13 (IVW:95% CI, 1.03–1.23), 1.12 (MR-Egger: 95%CI, 0.85–1.48), and 1.15 (WME:95%CI, 1.04–1.27) (*p* = 0.008). These results suggest that related factors did not affect the relationship between HF and CKD. The results are presented in Fig 4 (S11 Table).

### 3.2 Effect of CKD on HF

**Fig 5:**
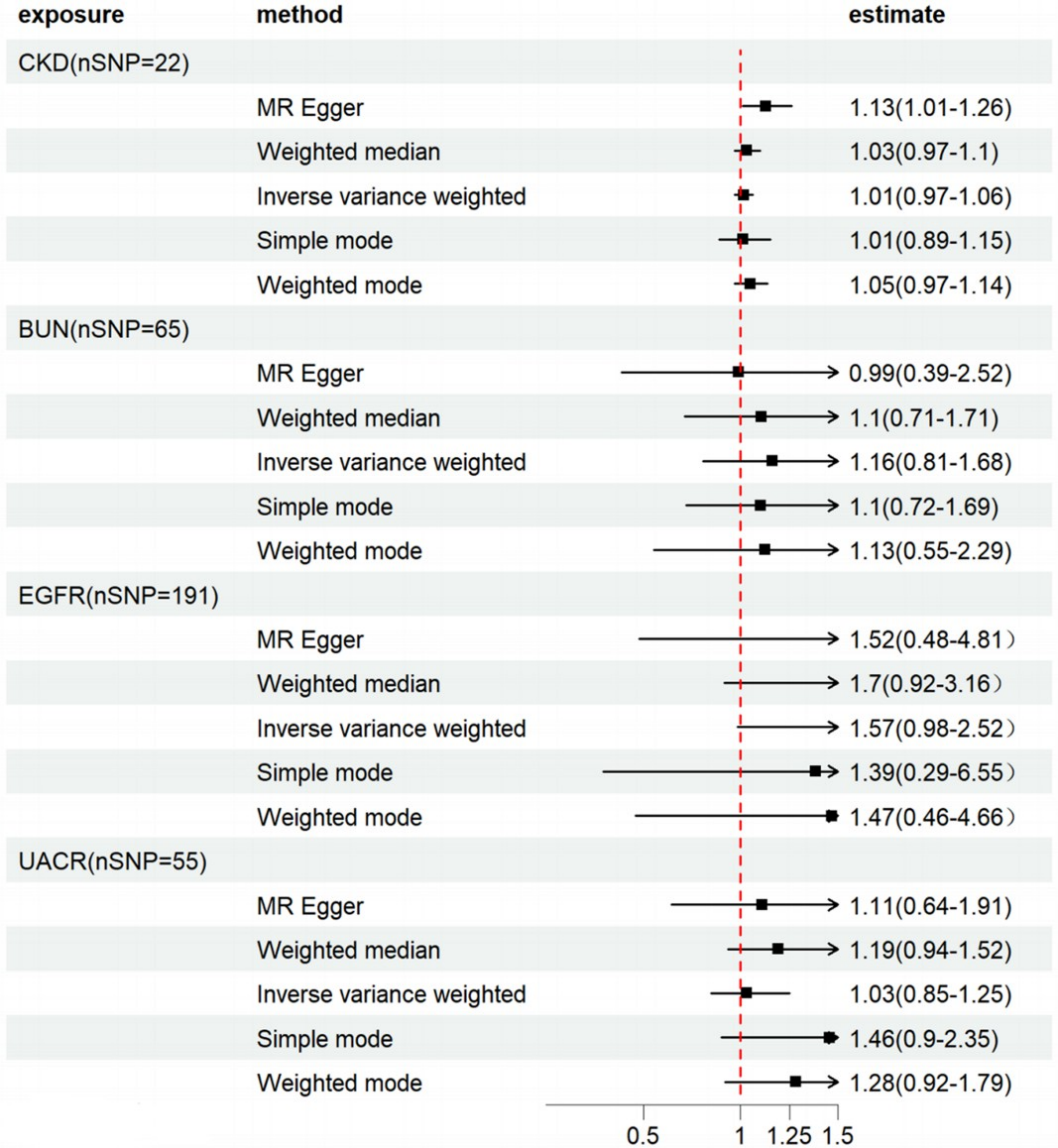
Forest plot of CKD effects on HF.

**Fig 6:**
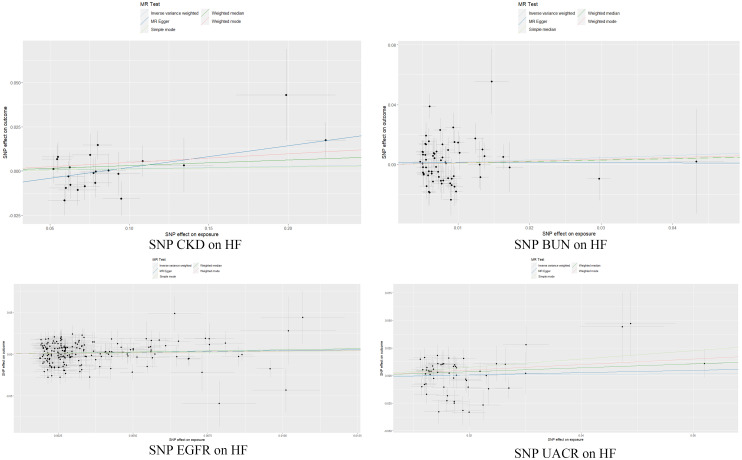
Map of intercept. The slope of each line corresponds to the estimated MR effect in different models.

In the reverse MR, CKD and kidney function was the exposure, and HF was the outcome. Final MR Analysis revealed 22, 65, 194, and 55 IVs that were associated with CKD, BUN, EGFR, and UACR, respectively. Multiple MR Analyses did not support the inference of CKD and renal function indexes on HF, as seen in Figs 5 and 6.

### 3.3 Sensitivity analysis

We performed multiple analyses to investigate the causal association between HF and CKD. Neither MR-Egger (intercept) nor MR-PRESSO methods detected any evidence of horizontal pleiotropy. We identified one outlier SNP and removed it using global cycle detection in MR-Presso (NbDistribution = 10000). Additionally, we identified six SNPs associated with known risk factors for CKD (hypertension and diabetes) through Phenoscanner and conducted MR analysis after deletion. The causal association between HF and CKD remained supported. Also, results from the "Leave-one-out" sensitivity analysis were similar to those when all SNPs were used in the IVW analysis. Furthermore, no significant impact on causal association estimates was observed after excluding any SNP (Specific resulting images are shown in the additional file).

## 4 Discussion

In this study, we used pooled GWAS data from Europeans to explore the connection between HF and CKD and renal function through a bidirectional two-sample MR analysis. The primary baseline data was derived from the HERMES Consortium meta-analysis, which identified 12 genetic variants in 11 genomic regions associated with HF. Data from a large-scale GWAS meta-analysis, which included 51,861 cases related to UACR and 41,395 cases concerning eGFR, BUN, and CKD, was used to investigate CKD and renal function.

Our results indicate that a genetically-predicted HF is linked to an elevated risk of CKD but not a higher risk of renal dysfunction. Moreover, we found that HF and CKD were causally linked, which is in line with earlier research when SNPs of known risk factors for CKD were disregarded. The primary MR results back the causal inference of HF for CKD. On the other hand, genetic predictors of CKD and renal function were not found to be connected to a heightened risk of HF. To our knowledge, this is the exploration using MR to analyze the connection between HF and CKD and how it affects renal function. We employed various techniques to identify and remove outliers and obtained consistent positive results. Additionally, multiple MR methods yielded the same results, implying the credibility of the findings.

Numerous investigations have explored the connection between HF and CKD. Damman et al. reported that worsening renal function (WRF) and CKD are prevalent in HF patients, with a prevalence of up to 32% and 23%, respectively, and are correlated with a heightened likelihood of mortality [31]. People with CKD face double the risk of death compared to the general population, with over 50% dying from cardiovascular disease [32]. Both diseases were linked to reduced survival when CKD was present, although the effect on mortality was more pronounced in the presence of CKD [33]. Deterioration of renal function during or after hospitalization is closely related to long-term prognosis. A retrospective analysis of the SOLVD and the PRIME II study showed that even if patients have normal left ventricular function and no signs of severe heart failure, having impaired renal function still decreases the chance of survival [34]. Smith et al. conducted a meta-analysis in 2006, which included 15 studies concerning renal impairment and its effect on the prognosis of HF, and found that any level of kidney damage was linked to a 56% higher risk of death [35].

The interplay between the heart and kidneys is intricate and bidirectional. Patients with chronic HF may observe a decline in their blood pressure and circulation, activating the sympathetic nervous system and the renin-angiotensin-aldosterone system as a way to compensate [36]. Angiotensin II (AII), resulting from RAAS activation, triggers the sympathetic nervous system, taking charge of the afferent and efferent arterioles of the glomeruli [37]. The sympathetic nervous system causes the vessels to constrict, decreasing the amount of blood flowing to the kidneys and reducing the GFR [38]. Prolonged hypoperfusion and hypoxia can heighten oxidative stress and inflammatory responses in the kidneys [39]. Furthermore, hypotension and organ hypoperfusion due to ATII in CHF increase AVP and EI production. EI is a protein that has the potential to cause inflammation, fibrosis, and a narrowing of blood vessels, and it maintains the inflammation in the kidneys by stimulating TGF-β and NF-κB [40]. The heart and kidneys have a close connection when it comes to acute cardio-renal syndrome (cardio-renal syndrome type 1) [41]. A reduced cardiac output, clinically referred to as "forward failure," can bring on tubular hypoxia and acute tubular necrosis because tubular cells are so sensitive to low oxygen levels [42]. Moreover, comorbidities such as diabetes may worsen this damage by causing chronic tubule hypoxia, inflammation, and tubule interstitial fibrosis [43,44]. It has been traditionally accepted that "forward failure" is a major contributor to renal dysfunction in the acute setting regarding heart-kidney interaction [45]. Therefore, the heart and kidneys can be affected by both short-term and long-term diseases, as they are closely related.

The correlation between HF and CKD is of great importance for preventing and treating both conditions. Unfortunately, the two-year survival rate for Medicare patients with HF and CKD is only 76%, compared to 93% for those without either disease [46]. For the past couple of decades, ACEI and ARBs have been used to treat HF and decreased ejection fraction and to deal with albuminuria in CKD [47]. Generally, the beginning of treatment with these drugs is usually accompanied by a slight, temporary decline in renal function due to the hemodynamic effect of these drugs on the pressure within the renal glomeruli.

## 5 Strengths and limitations

This research adopted MR to explore the causal link between HF and CKD in a European population. This method is beneficial in that it can identify the causal link between HF and the genetic risk of CKD in the same sample. Moreover, due to Mendelian inheritance’s second law, which ensures alleles are randomly assigned, it avoids bias and reverses causation. Further analysis involving HF, CKD, and other renal function-associated elements (EGFR, UACR, BUN) affirmed the positive causal relationship between HF and CKD.

This research had some constraints. For example, the genetic data was only gathered from people of European heritage. To ensure that any racial discrepancies are ruled out, more MR Studies should be conducted on other ethnicities, such as Asians and Africans, to determine a more accurate causal link between HF and CKD.

## 6 Conclusion

Our Bidirectional Two-Sample MR Study indicates that HF is causally linked to CKD in a European population. We strengthened our findings by using various MR methods and controlling for potential confounders. This study provides evidence for personalized treatment, drug development, and prevention of HF and CKD. Unraveling the causal relationship between HF and CKD can offer new avenues for personalized medicine. Subsequent research should investigate the possibility of treatment modalities tailored to particular patient subgroups to reduce the risk and progression of both diseases. In addition, this discovery offers valuable insights for the development of innovative pharmacological treatments. Future investigations should focus on exploring the molecular mechanisms linking HF and CKD to identify potential pharmaceutical targets, which could lead to developing more efficacious drugs to address both conditions. Furthermore, understanding the causal role of HF in the context of CKD can serve as a guide for future preventive measures. Preventing the occurrence of HF or managing HF effectively may reduce the risk of CKD. Further research should be conducted to examine if our findings can be applied to other populations and to explore the underlying molecular mechanisms of the heart-kidney interaction to identify potential therapeutic targets.

## Supporting information

S1 Table Instrumental variables of HF effect on CKD. (DOC)

S2 Table Instrumental variables of HF effect on BUN. (DOC)

S3 Table Instrumental variables of HF effect on EGFR. (DOC)

S4 Table Instrument variables of HF effect on UACR. (DOC)

S5 Table Instrument variables of CKD effect on HF. (DOC)

S6 Table Instrument variables of BUN effect on HF. (DOC)

S7 Table Instrument variables of EGFR effect on HF. (DOC)

S8 Table Instrument variables of UACR effect on HF. (DOC)

S9 Table MR estimates of the causal association between HF and Kidney Function and CKD. (DOC)

S10 Table MR estimates of the causal association between HF and Kidney Function and CKD(after MR-presso). (DOC)

S11 Table MR estimates of HF effects on CKD(Hypertension and Diabetes mellitus were excluded). (DOC)
