# Supplementary material for: Causal relationship from heart failure to kidney function and CKD: A bidirectional two-sample mendelian randomization study
Source: PLoS One. 2023 Dec 11;18(12):e0295532. doi: 10.1371/journal.pone.0295532 (PMC10712866; doi:10.1371/journal.pone.0295532)
Supplement: S1 Table — (DOC) [file pone.0295532.s001.doc]

**S1 Table. Instrumental variables of HF effect on CKD**

| SNP | effect_allele | other_allele | beta | eaf | se | pval |
| --- | --- | --- | --- | --- | --- | --- |
| rs10150022 | A | G | 0.0419 | 0.2924 | 0.0087 | 1.46E-06 |
| rs10459012 | A | C | 0.0458 | 0.2249 | 0.0095 | 1.43E-06 |
| rs10520390 | C | G | 0.0902 | 0.9467 | 0.0187 | 1.41E-06 |
| rs10846742 | A | G | -0.0506 | 0.8478 | 0.0111 | 5.15E-06 |
| rs10882816 | T | G | -0.0447 | 0.3267 | 0.0085 | 1.45E-07 |
| rs10938398 | A | G | 0.0389 | 0.4258 | 0.008 | 1.16E-06 |
| rs10952517 | A | T | -0.0394 | 0.3718 | 0.0085 | 3.56E-06 |
| rs11722972 | T | G | 0.0519 | 0.8244 | 0.0114 | 5.30E-06 |
| rs11745324 | A | G | -0.0528 | 0.2277 | 0.0095 | 2.73E-08 |
| rs117925145 | A | G | -0.1797 | 0.9829 | 0.0391 | 4.31E-06 |
| rs11874705 | A | G | -0.0469 | 0.7968 | 0.0098 | 1.70E-06 |
| rs12477245 | T | C | 0.1192 | 0.0313 | 0.0236 | 4.40E-07 |
| rs12805761 | A | T | 0.0672 | 0.7496 | 0.0139 | 1.33E-06 |
| rs12940636 | T | C | 0.0381 | 0.6507 | 0.0083 | 4.42E-06 |
| rs1510226 | T | C | -0.162 | 0.9807 | 0.0285 | 1.31E-08 |
| rs17483686 | A | T | -0.0489 | 0.6661 | 0.0095 | 2.64E-07 |
| rs17496249 | A | G | 0.0372 | 0.5452 | 0.0079 | 2.49E-06 |
| rs17617337 | T | C | -0.0561 | 0.2208 | 0.0095 | 3.52E-09 |
| rs1788761 | A | G | 0.0425 | 0.5121 | 0.009 | 2.33E-06 |
| rs186973337 | A | T | 0.0933 | 0.0521 | 0.0196 | 1.93E-06 |
| rs2634071 | T | C | 0.0923 | 0.1763 | 0.0101 | 6.33E-20 |
| rs2980858 | T | C | 0.04 | 0.3055 | 0.0086 | 3.30E-06 |
| rs35005436 | T | C | -0.0533 | 0.8468 | 0.0116 | 4.33E-06 |
| rs35054810 | A | G | 0.0725 | 0.0864 | 0.0143 | 3.98E-07 |
| rs4135240 | T | C | 0.0486 | 0.6589 | 0.0084 | 7.22E-09 |
| rs4376020 | A | T | -0.0612 | 0.1353 | 0.0123 | 6.50E-07 |
| rs4746140 | C | G | -0.0666 | 0.154 | 0.0109 | 9.96E-10 |
| rs4755717 | C | G | 0.0379 | 0.5872 | 0.008 | 2.16E-06 |
| rs55730499 | T | C | 0.1058 | 0.0694 | 0.0157 | 1.60E-11 |
| rs55751848 | C | G | 0.0425 | 0.7078 | 0.0089 | 1.79E-06 |
| rs55949718 | T | C | -0.0685 | 0.0878 | 0.0142 | 1.41E-06 |
| rs56094641 | A | G | -0.0454 | 0.5842 | 0.008 | 1.39E-08 |
| rs578065 | T | G | -0.0408 | 0.6462 | 0.0082 | 6.50E-07 |
| rs593467 | A | G | -0.0548 | 0.8688 | 0.0118 | 3.42E-06 |
| rs600038 | T | C | -0.0569 | 0.7909 | 0.0096 | 3.08E-09 |
| rs61733868 | T | C | 0.1057 | 0.9613 | 0.0216 | 9.90E-07 |
| rs660240 | T | C | -0.0611 | 0.2128 | 0.0097 | 3.00E-10 |
| rs6922885 | T | C | 0.0377 | 0.4565 | 0.008 | 2.45E-06 |
| rs72807031 | A | T | -0.0895 | 0.0748 | 0.0181 | 7.62E-07 |
| rs72844714 | A | C | 0.0559 | 0.1647 | 0.0121 | 3.84E-06 |
| rs73200714 | A | G | -0.055 | 0.1475 | 0.0118 | 3.15E-06 |
| rs7559452 | A | G | -0.0468 | 0.819 | 0.0102 | 4.47E-06 |
| rs76117960 | T | C | -0.0528 | 0.8523 | 0.0113 | 2.97E-06 |
| rs7766436 | T | C | 0.04 | 0.2789 | 0.0086 | 3.30E-06 |
| rs7859727 | T | C | 0.0623 | 0.4775 | 0.0078 | 1.38E-15 |
| rs80087882 | A | G | 0.0609 | 0.1097 | 0.0125 | 1.10E-06 |
| rs8017852 | A | C | -0.0554 | 0.1328 | 0.012 | 3.90E-06 |
| rs8081247 | A | G | -0.0487 | 0.8053 | 0.0098 | 6.72E-07 |
| rs9815816 | T | C | -0.0479 | 0.8034 | 0.0099 | 1.31E-06 |
| rs994980 | T | C | 0.0375 | 0.6086 | 0.0081 | 3.66E-06 |
| Outlier instrumental variables(method:MR-Presso ,NbDistribution = 10000) | | | | | | |
| rs4746140 | C | G | -0.0666 | 0.154 | 0.0109 | 9.96E-10 |
| Instrumental variables related with Hypertension and Diabetes | | | | | | |
| rs11745324 | A | G | -0.0528 | 0.2277 | 0.0095 | 2.73E-08 |
| rs17617337 | T | C | -0.0561 | 0.2208 | 0.0095 | 3.52E-09 |
| rs35005436 | T | C | -0.0533 | 0.8468 | 0.0116 | 4.33E-06 |
| rs56094641 | A | G | -0.0454 | 0.5842 | 0.008 | 1.39E-08 |
| rs600038 | T | C | -0.0569 | 0.7909 | 0.0096 | 3.08E-09 |
| rs73200714 | A | G | -0.055 | 0.1475 | 0.0118 | 3.15E-06 |
