# Supplementary material for: Causal relationship from heart failure to kidney function and CKD: A bidirectional two-sample mendelian randomization study
Source: PLoS One. 2023 Dec 11;18(12):e0295532. doi: 10.1371/journal.pone.0295532 (PMC10712866; doi:10.1371/journal.pone.0295532)
Supplement: S5 Table — (DOC) [file pone.0295532.s005.doc]

**S5 Table. Instrumental variables of CKD effect on HF**

| SNP | effect_allele | other_allele | eaf | beta | se | pval |
| --- | --- | --- | --- | --- | --- | --- |
| rs10224002 | A | G | -0.1083 | 0.72 | 0.0102 | 2.65E-26 |
| rs1049518 | A | G | 0.0788 | 0.38 | 0.0094 | 5.42E-17 |
| rs11761603 | T | C | -0.0674 | 0.3 | 0.0119 | 1.35E-08 |
| rs12205178 | A | G | 0.0931 | 0.12 | 0.014 | 3.09E-11 |
| rs13391258 | T | C | -0.06 | 0.24 | 0.0108 | 2.74E-08 |
| rs1458038 | T | C | -0.059 | 0.31 | 0.01 | 4.21E-09 |
| rs17730281 | A | G | -0.0869 | 0.23 | 0.011 | 2.68E-15 |
| rs187355703 | C | G | -0.1987 | 0.98 | 0.0312 | 1.80E-10 |
| rs1889937 | A | G | -0.0624 | 0.63 | 0.01 | 5.15E-10 |
| rs2484639 | A | G | -0.0774 | 0.51 | 0.0092 | 2.95E-17 |
| rs2580350 | A | G | 0.055 | 0.55 | 0.0098 | 1.69E-08 |
| rs35716097 | T | C | 0.0785 | 0.32 | 0.0105 | 8.20E-14 |
| rs3925584 | T | C | 0.08 | 0.56 | 0.0092 | 4.68E-18 |
| rs4871907 | A | C | -0.0628 | 0.55 | 0.0097 | 9.91E-11 |
| rs62300825 | A | G | -0.0949 | 0.2 | 0.0116 | 2.63E-16 |
| rs700221 | A | G | -0.0719 | 0.59 | 0.0098 | 2.19E-13 |
| rs7178881 | A | C | -0.0544 | 0.41 | 0.0092 | 4.14E-09 |
| rs77713116 | C | G | -0.0752 | 0.65 | 0.0116 | 1.03E-10 |
| rs77924615 | A | G | -0.2237 | 0.2 | 0.0128 | 6.38E-69 |
| rs7908590 | C | G | -0.1343 | 0.93 | 0.0188 | 8.99E-13 |
| rs881858 | A | G | 0.0616 | 0.7 | 0.0101 | 1.19E-09 |
| rs9474801 | A | G | 0.0522 | 0.34 | 0.0096 | 4.61E-08 |
