# Supplementary material for: Causal relationship from heart failure to kidney function and CKD: A bidirectional two-sample mendelian randomization study
Source: PLoS One. 2023 Dec 11;18(12):e0295532. doi: 10.1371/journal.pone.0295532 (PMC10712866; doi:10.1371/journal.pone.0295532)
Supplement: S7 Table — (DOC) [file pone.0295532.s007.doc]

**S7 Table. Instrumental variables of EGFR effect on HF**

| SNP | effect_allele | other_allele | beta | eaf | se | pval |
| --- | --- | --- | --- | --- | --- | --- |
| rs10086569 | T | C | 0.002774 | 0.24 | 0.0004029 | 5.73E-12 |
| rs10122824 | T | G | -0.002391 | 0.34 | 0.000379 | 2.84E-10 |
| rs10224002 | A | G | 0.006845 | 0.72 | 0.000398 | 2.74E-66 |
| rs1028455 | A | T | 0.002062 | 0.33 | 0.0003669 | 1.90E-08 |
| rs10430743 | T | G | 0.002525 | 0.43 | 0.0003458 | 2.86E-13 |
| rs1047891 | A | C | -0.006515 | 0.31 | 0.0003852 | 3.59E-64 |
| rs10498755 | T | C | -0.004914 | 0.083 | 0.0006297 | 6.04E-15 |
| rs10838702 | T | G | -0.002315 | 0.38 | 0.0003542 | 6.31E-11 |
| rs10846157 | A | C | -0.003612 | 0.81 | 0.0004367 | 1.34E-16 |
| rs10851885 | A | G | 0.004972 | 0.76 | 0.0004077 | 3.28E-34 |
| rs10887903 | A | G | 0.001887 | 0.53 | 0.0003423 | 3.52E-08 |
| rs10964603 | T | C | -0.002484 | 0.78 | 0.0004303 | 7.75E-09 |
| rs10994860 | T | C | 0.003891 | 0.19 | 0.000446 | 2.70E-18 |
| rs11062167 | A | G | -0.004179 | 0.53 | 0.0003444 | 7.08E-34 |
| rs11071738 | T | C | -0.00249 | 0.53 | 0.0003452 | 5.49E-13 |
| rs11227260 | T | G | -0.00322 | 0.35 | 0.0003608 | 4.47E-19 |
| rs112545201 | T | C | -0.004215 | 0.13 | 0.0005066 | 8.79E-17 |
| rs11261022 | A | C | -0.002713 | 0.36 | 0.0003583 | 3.67E-14 |
| rs112880707 | T | C | 0.005647 | 0.11 | 0.0005731 | 6.69E-23 |
| rs113572081 | C | G | -0.002741 | 0.14 | 0.0005026 | 4.92E-08 |
| rs1153855 | C | G | 0.008636 | 0.62 | 0.0003524 | 1.23E-132 |
| rs115926813 | A | G | -0.005442 | 0.04 | 0.0008957 | 1.24E-09 |
| rs11644400 | T | C | -0.002963 | 0.85 | 0.0004863 | 1.11E-09 |
| rs11657044 | T | C | -0.00755 | 0.17 | 0.0004621 | 5.34E-60 |
| rs11694902 | A | G | 0.004132 | 0.14 | 0.000503 | 2.14E-16 |
| rs117113238 | A | G | 0.003939 | 0.095 | 0.0006099 | 1.06E-10 |
| rs11753995 | A | G | 0.003358 | 0.17 | 0.000464 | 4.58E-13 |
| rs11784052 | T | C | 0.002726 | 0.46 | 0.000352 | 9.78E-15 |
| rs11794652 | A | G | -0.002759 | 0.16 | 0.0004804 | 9.34E-09 |
| rs11856829 | T | C | 0.002692 | 0.49 | 0.0003674 | 2.38E-13 |
| rs11951093 | A | G | -0.005581 | 0.42 | 0.0003593 | 2.05E-54 |
| rs12163971 | A | C | -0.003229 | 0.16 | 0.0004663 | 4.33E-12 |
| rs12207180 | A | T | -0.008522 | 0.12 | 0.0005373 | 1.21E-56 |
| rs1268176 | A | G | 0.002728 | 0.34 | 0.0003643 | 7.05E-14 |
| rs12736457 | C | G | 0.0056 | 0.87 | 0.0005227 | 8.79E-27 |
| rs12920176 | A | C | -0.002618 | 0.59 | 0.0003573 | 2.35E-13 |
| rs12940987 | A | G | -0.00428 | 0.77 | 0.0004132 | 3.84E-25 |
| rs12989250 | A | G | -0.00256 | 0.31 | 0.0003777 | 1.23E-11 |
| rs13047277 | T | C | 0.002158 | 0.72 | 0.0003955 | 4.86E-08 |
| rs13146355 | A | G | -0.007419 | 0.44 | 0.0003463 | 8.31E-102 |
| rs13157326 | A | G | -0.002706 | 0.48 | 0.0003877 | 2.95E-12 |
| rs13230509 | C | G | -0.005525 | 0.69 | 0.000434 | 4.04E-37 |
| rs132641 | A | G | -0.002661 | 0.16 | 0.0004681 | 1.30E-08 |
| rs1377164 | T | C | 0.003357 | 0.21 | 0.0004181 | 9.84E-16 |
| rs1397764 | A | G | 0.004656 | 0.28 | 0.000384 | 7.83E-34 |
| rs140179699 | A | G | 0.007306 | 0.95 | 0.0010769 | 1.16E-11 |
| rs1458038 | T | C | 0.003197 | 0.3 | 0.0003795 | 3.60E-17 |
| rs1509117 | A | T | 0.002513 | 0.3 | 4.00E-04 | 3.33E-10 |
| rs1548945 | T | C | 0.003679 | 0.41 | 0.0003591 | 1.27E-24 |
| rs1569011 | A | G | 0.001954 | 0.44 | 0.0003467 | 1.73E-08 |
| rs1585499 | T | C | -0.002956 | 0.45 | 0.0003541 | 7.00E-17 |
| rs1595810 | A | G | -0.002371 | 0.2 | 0.0004332 | 4.40E-08 |
| rs1643471 | T | C | 0.002273 | 0.54 | 0.0003649 | 4.68E-10 |
| rs168505 | T | C | -0.002675 | 0.4 | 0.0003502 | 2.18E-14 |
| rs16930370 | T | C | 0.004084 | 0.82 | 0.0004528 | 1.87E-19 |
| rs1719934 | A | G | 0.002806 | 0.54 | 0.0003444 | 3.73E-16 |
| rs17696736 | A | G | 0.002029 | 0.57 | 0.0003538 | 9.72E-09 |
| rs1819008 | T | C | -0.001922 | 0.55 | 0.0003449 | 2.49E-08 |
| rs187355703 | C | G | 0.010107 | 0.97 | 0.0011431 | 9.45E-19 |
| rs1883991 | A | C | -0.003184 | 0.69 | 0.0003756 | 2.33E-17 |
| rs1887252 | C | G | -0.002889 | 0.64 | 0.0003584 | 7.45E-16 |
| rs1910738 | T | G | 0.002278 | 0.71 | 0.0003992 | 1.15E-08 |
| rs1913641 | T | G | -0.002001 | 0.48 | 0.0003418 | 4.77E-09 |
| rs1994887 | A | C | -0.002361 | 0.28 | 0.0003954 | 2.34E-09 |
| rs2039424 | A | G | 0.004828 | 0.62 | 0.0003613 | 9.75E-41 |
| rs2068888 | A | G | -0.002622 | 0.45 | 0.0003496 | 6.31E-14 |
| rs2071047 | A | G | 0.001999 | 0.41 | 0.0003498 | 1.10E-08 |
| rs2074204 | T | C | -0.002487 | 0.26 | 0.0003918 | 2.18E-10 |
| rs2145166 | A | G | -0.003283 | 0.16 | 0.0005784 | 1.38E-08 |
| rs2156664 | T | C | -0.002144 | 0.27 | 0.0003897 | 3.76E-08 |
| rs223308 | A | G | -0.002702 | 0.52 | 0.0003425 | 3.00E-15 |
| rs2235826 | A | T | -0.003281 | 0.81 | 0.000452 | 3.94E-13 |
| rs2244237 | T | G | 0.00268 | 0.22 | 0.0004128 | 8.43E-11 |
| rs2252281 | T | C | 0.004068 | 0.61 | 0.0003588 | 8.47E-30 |
| rs233438 | A | G | 0.004284 | 0.81 | 0.0004414 | 2.84E-22 |
| rs2365286 | A | G | -0.003347 | 0.74 | 0.0003943 | 2.08E-17 |
| rs2412608 | T | C | 0.003082 | 0.49 | 0.0003554 | 4.31E-18 |
| rs2490391 | A | C | -0.002497 | 0.46 | 0.0003461 | 5.46E-13 |
| rs2509851 | A | C | 0.002132 | 0.63 | 0.0003535 | 1.62E-09 |
| rs2608915 | A | G | 0.002527 | 0.78 | 0.0004233 | 2.38E-09 |
| rs2634675 | A | G | 0.002807 | 0.46 | 0.0003889 | 5.30E-13 |
| rs267738 | T | G | -0.005007 | 0.79 | 0.0004211 | 1.33E-32 |
| rs27879 | A | C | -0.001974 | 0.6 | 0.000351 | 1.86E-08 |
| rs2792796 | T | C | -0.002084 | 0.61 | 0.0003518 | 3.14E-09 |
| rs281380 | T | C | -0.002217 | 0.63 | 0.0003691 | 1.90E-09 |
| rs2823139 | A | G | -0.002714 | 0.34 | 0.0003648 | 1.01E-13 |
| rs2834321 | A | G | -0.002685 | 0.18 | 0.0004496 | 2.34E-09 |
| rs2954017 | T | C | 0.002635 | 0.46 | 0.000397 | 3.18E-11 |
| rs3018667 | A | G | -0.002375 | 0.32 | 0.000369 | 1.23E-10 |
| rs303937 | A | T | 0.002709 | 0.41 | 0.0003563 | 2.90E-14 |
| rs3111316 | A | G | -0.001923 | 0.59 | 0.0003525 | 4.89E-08 |
| rs3134605 | T | C | 0.003285 | 0.8 | 0.0004497 | 2.77E-13 |
| rs325442 | A | G | 0.002074 | 0.4 | 0.0003498 | 3.02E-09 |
| rs34117451 | T | C | -0.002594 | 0.17 | 0.0004583 | 1.52E-08 |
| rs35004449 | T | G | 0.002701 | 0.27 | 0.0003859 | 2.56E-12 |
| rs35072105 | A | G | -0.002108 | 0.55 | 0.0003513 | 1.97E-09 |
| rs35472707 | T | C | -0.007542 | 0.05 | 0.0008293 | 9.53E-20 |
| rs35629566 | C | G | 0.002976 | 0.83 | 0.0004801 | 5.73E-10 |
| rs363092 | A | C | -0.002179 | 0.42 | 0.0003513 | 5.55E-10 |
| rs3731906 | T | C | -0.002956 | 0.67 | 0.0003731 | 2.30E-15 |
| rs3744139 | T | G | 0.00228 | 0.66 | 0.0003631 | 3.39E-10 |
| rs3757387 | T | C | 0.002914 | 0.55 | 0.0003555 | 2.48E-16 |
| rs3791221 | A | G | 0.002138 | 0.65 | 0.0003604 | 3.00E-09 |
| rs3793805 | A | G | -0.002008 | 0.57 | 0.0003506 | 1.03E-08 |
| rs3795503 | T | C | 0.002156 | 0.33 | 0.0003808 | 1.51E-08 |
| rs3797537 | A | G | 0.00212 | 0.71 | 0.0003768 | 1.86E-08 |
| rs3812036 | T | C | -0.006869 | 0.26 | 0.0004059 | 3.19E-64 |
| rs3814828 | A | G | 0.002211 | 0.38 | 0.0003693 | 2.13E-09 |
| rs3822939 | A | G | -0.002807 | 0.46 | 0.0003436 | 3.08E-16 |
| rs3845534 | A | G | -0.001903 | 0.49 | 0.0003463 | 3.91E-08 |
| rs3850625 | A | G | 0.004806 | 0.12 | 0.0005529 | 3.57E-18 |
| rs3905668 | A | G | -0.00255 | 0.72 | 0.000383 | 2.81E-11 |
| rs3925584 | T | C | -0.005472 | 0.55 | 0.0003463 | 3.01E-56 |
| rs396341 | T | C | 0.002973 | 0.26 | 0.0003876 | 1.72E-14 |
| rs3993747 | A | G | -0.002169 | 0.64 | 0.0003648 | 2.73E-09 |
| rs407102 | T | C | 0.003086 | 0.7 | 0.0003789 | 3.80E-16 |
| rs4072824 | A | C | 0.00227 | 0.67 | 0.000394 | 8.38E-09 |
| rs41284816 | T | G | -0.007874 | 0.026 | 0.0012304 | 1.56E-10 |
| rs4410790 | T | C | -0.002286 | 0.37 | 0.000359 | 1.92E-10 |
| rs4566 | T | G | 0.002033 | 0.61 | 0.000355 | 1.03E-08 |
| rs4567937 | A | G | -0.003186 | 0.32 | 0.0003694 | 6.40E-18 |
| rs4656220 | T | C | 0.002143 | 0.37 | 0.0003551 | 1.60E-09 |
| rs4668134 | A | T | -0.010187 | 0.98 | 0.0015925 | 1.58E-10 |
| rs4788809 | A | G | -0.002116 | 0.37 | 0.0003548 | 2.46E-09 |
| rs4794814 | A | G | -0.005875 | 0.75 | 0.0003992 | 5.03E-49 |
| rs4808154 | T | C | 0.002596 | 0.71 | 0.0004497 | 7.77E-09 |
| rs4820324 | C | G | -0.002343 | 0.58 | 0.0003507 | 2.39E-11 |
| rs4836732 | T | C | 0.002518 | 0.53 | 0.0003481 | 4.69E-13 |
| rs499600 | T | G | -0.003703 | 0.15 | 0.0004776 | 8.93E-15 |
| rs544169 | A | G | 0.00237 | 0.74 | 0.0003878 | 9.98E-10 |
| rs55759218 | A | G | -0.003904 | 0.27 | 0.0003869 | 6.09E-24 |
| rs55808581 | C | G | 0.002727 | 0.17 | 0.0004658 | 4.75E-09 |
| rs55938024 | A | G | -0.006457 | 0.12 | 0.0006051 | 1.37E-26 |
| rs6011067 | C | G | 0.003551 | 0.92 | 0.0006475 | 4.16E-08 |
| rs6029632 | A | G | 0.001978 | 0.4 | 0.0003489 | 1.43E-08 |
| rs6088528 | A | G | -0.003291 | 0.5 | 0.0003435 | 9.61E-22 |
| rs6127099 | A | T | -0.005128 | 0.72 | 0.0004055 | 1.17E-36 |
| rs6135224 | A | G | -0.002023 | 0.69 | 0.0003685 | 4.04E-08 |
| rs61927768 | A | G | -0.002284 | 0.29 | 0.0003915 | 5.36E-09 |
| rs623834 | T | C | -0.002152 | 0.58 | 0.0003625 | 2.90E-09 |
| rs62394289 | A | G | 0.003651 | 0.12 | 0.0005241 | 3.23E-12 |
| rs62432759 | A | G | -0.002494 | 0.78 | 0.0004317 | 7.56E-09 |
| rs62491533 | T | C | -0.002741 | 0.83 | 0.0004576 | 2.11E-09 |
| rs6458868 | T | C | -0.002128 | 0.65 | 0.0003602 | 3.50E-09 |
| rs6481598 | C | G | 0.002313 | 0.78 | 0.0004204 | 3.77E-08 |
| rs6546869 | A | G | 0.006087 | 0.22 | 0.0004159 | 1.66E-48 |
| rs6555317 | A | G | 0.002385 | 0.69 | 0.0004108 | 6.45E-09 |
| rs66473811 | T | C | 0.003072 | 0.84 | 0.000483 | 2.02E-10 |
| rs6667182 | T | C | -0.004284 | 0.32 | 0.00043 | 2.22E-23 |
| rs67216675 | T | G | 0.002222 | 0.32 | 0.0003692 | 1.75E-09 |
| rs67571561 | T | C | 0.006393 | 0.96 | 0.000892 | 7.64E-13 |
| rs6921580 | C | G | 0.002735 | 0.41 | 0.0003562 | 1.61E-14 |
| rs6948759 | T | C | -0.002577 | 0.21 | 0.000422 | 1.01E-09 |
| rs6971211 | T | C | -0.002867 | 0.41 | 0.0003635 | 3.10E-15 |
| rs700753 | C | G | 0.003295 | 0.34 | 0.0003613 | 7.50E-20 |
| rs7084764 | A | G | 0.002618 | 0.5 | 0.0003444 | 2.92E-14 |
| rs71606723 | A | T | 0.002893 | 0.76 | 0.0004052 | 9.26E-13 |
| rs7185391 | T | G | -0.002646 | 0.29 | 0.00039 | 1.15E-11 |
| rs7188071 | T | C | 0.002449 | 0.36 | 0.0003587 | 8.62E-12 |
| rs7203398 | A | C | 0.002729 | 0.73 | 0.0003907 | 2.86E-12 |
| rs72714330 | T | C | 0.003749 | 0.12 | 0.0005499 | 9.20E-12 |
| rs72995641 | A | G | -0.002592 | 0.2 | 0.0004279 | 1.39E-09 |
| rs73116829 | A | G | -0.004256 | 0.11 | 0.0005771 | 1.64E-13 |
| rs7326821 | A | G | 0.002569 | 0.83 | 0.0004673 | 3.82E-08 |
| rs736820 | A | G | -0.002147 | 0.37 | 0.0003676 | 5.27E-09 |
| rs7514450 | T | C | 0.002222 | 0.43 | 0.0003467 | 1.48E-10 |
| rs75267082 | A | T | 0.003397 | 0.89 | 0.0005581 | 1.15E-09 |
| rs7543734 | C | G | 0.00312 | 0.2 | 0.000482 | 9.58E-11 |
| rs7565830 | A | G | -0.002225 | 0.72 | 0.0003867 | 8.77E-09 |
| rs7592697 | T | C | -0.002036 | 0.65 | 0.0003681 | 3.18E-08 |
| rs7667050 | T | C | 0.002031 | 0.47 | 0.0003423 | 2.98E-09 |
| rs7719960 | A | G | 0.00259 | 0.23 | 0.000417 | 5.24E-10 |
| rs77924615 | A | G | 0.009576 | 0.2 | 0.0004519 | 1.21E-99 |
| rs780094 | T | C | 0.004601 | 0.38 | 0.0003565 | 4.16E-38 |
| rs78444298 | A | G | -0.010669 | 0.019 | 0.0014001 | 2.53E-14 |
| rs78614739 | T | C | 0.002588 | 0.17 | 0.0004616 | 2.06E-08 |
| rs78660602 | A | G | -0.005728 | 0.9 | 0.0005961 | 7.39E-22 |
| rs78986840 | T | C | -0.004479 | 0.94 | 0.0007392 | 1.37E-09 |
| rs79346194 | A | G | -0.002146 | 0.69 | 0.0003767 | 1.22E-08 |
| rs7974833 | T | C | -0.003224 | 0.76 | 0.0004104 | 3.97E-15 |
| rs79760705 | T | G | 0.005609 | 0.11 | 0.0005512 | 2.55E-24 |
| rs8028182 | T | G | -0.002591 | 0.19 | 0.000447 | 6.74E-09 |
| rs80282103 | A | T | 0.008084 | 0.92 | 0.0006333 | 2.58E-37 |
| rs8050794 | T | C | 0.002203 | 0.29 | 0.0003928 | 2.03E-08 |
| rs807624 | T | G | 0.003376 | 0.34 | 0.0003633 | 1.53E-20 |
| rs8101667 | T | C | 0.005007 | 0.33 | 0.0003625 | 2.19E-43 |
| rs881858 | A | G | -0.005595 | 0.7 | 0.0003776 | 1.15E-49 |
| rs929934 | T | C | 0.001985 | 0.43 | 0.0003546 | 2.16E-08 |
| rs9375702 | T | C | 0.002522 | 0.69 | 0.0003729 | 1.34E-11 |
| rs9419939 | A | G | 0.002595 | 0.2 | 0.0004445 | 5.31E-09 |
| rs9807656 | T | C | -0.003437 | 0.9 | 0.0005831 | 3.76E-09 |
| rs9838792 | A | G | 0.00314 | 0.39 | 0.0003521 | 4.76E-19 |
| rs9868185 | A | G | 0.002653 | 0.54 | 0.0003451 | 1.50E-14 |
| rs9894634 | T | C | -0.002107 | 0.6 | 0.0003497 | 1.69E-09 |
| Outlier instrumental variables(method:MR-Presso ,NbDistribution = 10000) | | | | | | |
| rs17696736 | A | G | 0.002029 | 0.57 | 0.0003538 | 9.72E-09 |
| rs2954017 | T | C | 0.002635 | 0.46 | 0.000397 | 3.18E-11 |
| rs9894634 | T | C | -0.002107 | 0.6 | 0.0003497 | 1.69E-09 |
