# Supplementary material for: Causal relationship from heart failure to kidney function and CKD: A bidirectional two-sample mendelian randomization study
Source: PLoS One. 2023 Dec 11;18(12):e0295532. doi: 10.1371/journal.pone.0295532 (PMC10712866; doi:10.1371/journal.pone.0295532)
Supplement: S8 Table — (DOC) [file pone.0295532.s008.doc]

**S8 Table. Instrumental variables of UACR effect on HF**

| SNP | effect_allele | other_allele | beta | eaf | se | pval |
| --- | --- | --- | --- | --- | --- | --- |
| rs10023335 | T | C | 0.01439 | 0.5932 | 0.002017 | 9.72E-13 |
| rs10207567 | C | G | 0.0193712 | 0.8151 | 0.002552 | 3.18E-14 |
| rs1047891 | A | C | -0.0189908 | 0.3148 | 0.0021754 | 2.55E-18 |
| rs1057868 | T | C | 0.0121717 | 0.2846 | 0.0021985 | 3.09E-08 |
| rs1086899 | T | C | -0.0123703 | 0.2903 | 0.0022318 | 2.98E-08 |
| rs11078597 | T | C | -0.0159921 | 0.813 | 0.0025948 | 7.13E-10 |
| rs112607182 | T | C | 0.0301346 | 0.0751 | 0.0041018 | 2.03E-13 |
| rs113139575 | C | G | -0.0248385 | 0.9366 | 0.004086 | 1.21E-09 |
| rs11659764 | A | T | 0.0300782 | 0.0527 | 0.0044753 | 1.81E-11 |
| rs11709284 | A | G | 0.0112451 | 0.5589 | 0.0019991 | 1.86E-08 |
| rs11912350 | T | C | -0.0130071 | 0.7582 | 0.002332 | 2.44E-08 |
| rs12714144 | A | T | 0.0224637 | 0.8733 | 0.0029873 | 5.49E-14 |
| rs12790943 | T | C | 0.0136538 | 0.422 | 0.0020126 | 1.17E-11 |
| rs1309546 | T | C | 0.0123644 | 0.5515 | 0.0019965 | 5.90E-10 |
| rs13132085 | A | G | -0.0127622 | 0.2893 | 0.0021969 | 6.28E-09 |
| rs1337526 | A | G | -0.027088 | 0.1983 | 0.0024882 | 1.34E-27 |
| rs144135542 | T | C | 0.0145616 | 0.1966 | 0.0026319 | 3.15E-08 |
| rs146311723 | T | C | -0.0154168 | 0.8235 | 0.0026582 | 6.64E-09 |
| rs15052 | T | C | 0.0173241 | 0.8253 | 0.0027287 | 2.17E-10 |
| rs162890 | T | C | 0.0134553 | 0.3317 | 0.002176 | 6.27E-10 |
| rs16864515 | A | C | -0.0188738 | 0.0965 | 0.0033621 | 1.98E-08 |
| rs1688031 | T | C | -0.019485 | 0.142 | 0.002893 | 1.64E-11 |
| rs17035646 | A | G | 0.0120302 | 0.3398 | 0.0021185 | 1.36E-08 |
| rs17158386 | A | G | 0.019817 | 0.2584 | 0.0023357 | 2.17E-17 |
| rs17343073 | A | T | -0.0619874 | 0.8958 | 0.003253 | 5.94E-81 |
| rs2068888 | A | G | -0.0124266 | 0.4515 | 0.0020029 | 5.50E-10 |
| rs2277537 | A | G | 0.0149652 | 0.597 | 0.0020316 | 1.76E-13 |
| rs2433611 | A | C | -0.0175444 | 0.2594 | 0.0022635 | 9.11E-15 |
| rs2470893 | T | C | 0.0230342 | 0.3259 | 0.0021538 | 1.08E-26 |
| rs2601006 | T | C | -0.015452 | 0.343 | 0.0020932 | 1.56E-13 |
| rs2880119 | A | C | -0.0164051 | 0.8582 | 0.0028558 | 9.22E-09 |
| rs2954021 | A | G | 0.0148457 | 0.4915 | 0.0019821 | 6.89E-14 |
| rs34257409 | T | G | 0.0160895 | 0.4038 | 0.0020197 | 1.63E-15 |
| rs35572189 | A | G | -0.0119865 | 0.3641 | 0.0021641 | 3.05E-08 |
| rs35692677 | A | G | -0.016352 | 0.1863 | 0.0025946 | 2.93E-10 |
| rs3734692 | A | T | -0.0177228 | 0.6908 | 0.0021814 | 4.49E-16 |
| rs3850625 | A | G | 0.0177182 | 0.1187 | 0.0031206 | 1.36E-08 |
| rs4410790 | T | C | -0.0219239 | 0.3689 | 0.0020613 | 2.03E-26 |
| rs45437393 | T | C | 0.0488045 | 0.0192 | 0.0080398 | 1.28E-09 |
| rs4641276 | T | C | -0.0128406 | 0.2455 | 0.0023308 | 3.61E-08 |
| rs4665972 | T | C | 0.0173726 | 0.3953 | 0.0020777 | 6.20E-17 |
| rs4738817 | A | G | -0.0115306 | 0.4535 | 0.0019942 | 7.39E-09 |
| rs4899263 | A | G | -0.0136243 | 0.5302 | 0.0020211 | 1.57E-11 |
| rs56336142 | T | C | -0.0173069 | 0.7876 | 0.002439 | 1.29E-12 |
| rs6535594 | A | G | 0.0143694 | 0.4979 | 0.0019939 | 5.74E-13 |
| rs67339103 | A | G | 0.0172135 | 0.2163 | 0.0024569 | 2.45E-12 |
| rs677888 | T | G | -0.0143186 | 0.7598 | 0.0023247 | 7.30E-10 |
| rs6998967 | A | G | -0.0152428 | 0.1664 | 0.0026868 | 1.40E-08 |
| rs7115200 | T | G | -0.0122547 | 0.5605 | 0.0020438 | 2.02E-09 |
| rs73065147 | T | C | -0.0264326 | 0.9306 | 0.0039274 | 1.69E-11 |
| rs7597336 | A | G | -0.0199876 | 0.8726 | 0.0029934 | 2.43E-11 |
| rs76027714 | A | G | 0.0234446 | 0.9238 | 0.0038402 | 1.03E-09 |
| rs7812843 | A | G | -0.0116995 | 0.501 | 0.0019827 | 3.62E-09 |
| rs78444298 | A | G | -0.0473471 | 0.0192 | 0.0075067 | 2.84E-10 |
| rs838142 | A | G | 0.017184 | 0.7203 | 0.0023041 | 8.78E-14 |
| rs988712 | T | G | -0.0131747 | 0.2363 | 0.0023395 | 1.79E-08 |
| Outlier instrumental variables(method:MR-Presso ,NbDistribution = 10000) | | | | | | |
| rs2954021 | A | G | 0.0148457 | 0.4915 | 0.0019821 | 6.89E-14 |
