# Supplementary material for: Causal relationship from heart failure to kidney function and CKD: A bidirectional two-sample mendelian randomization study
Source: PLoS One. 2023 Dec 11;18(12):e0295532. doi: 10.1371/journal.pone.0295532 (PMC10712866; doi:10.1371/journal.pone.0295532)
Supplement: S9 Table — (DOC) [file pone.0295532.s009.doc]

**S9 Table. MR estimates of the causal association between HF and Kidney Function and CKD**

| MR estimates | method | nsnp | beta | se | pval |
| --- | --- | --- | --- | --- | --- |
| BUN on HF | MR Egger | 68 | -0.069172021 | 0.537021815 | 0.897902299 |
|  | Weighted median | 68 | 0.159068142 | 0.222031396 | 0.473730909 |
|  | IVW | 68 | 0.283269276 | 0.207802469 | 0.172830176 |
|  | Simple mode | 68 | -0.495964904 | 0.543201499 | 0.36449518 |
|  | Weighted mode | 68 | 0.134967069 | 0.361716557 | 0.710230099 |
| CKD on HF | MR Egger | 22 | 0.12211002 | 0.05683696 | 0.044109438 |
|  | Weighted median | 22 | 0.031340939 | 0.031961899 | 0.326803924 |
|  | IVW | 22 | 0.013390174 | 0.023815395 | 0.573946617 |
|  | Simple mode | 22 | 0.011519583 | 0.066504589 | 0.864141261 |
|  | Weighted mode | 22 | 0.048744247 | 0.040723891 | 0.244666118 |
| EGFR on HF | MR Egger | 194 | 0.624395904 | 0.629825546 | 0.32274822 |
|  | Weighted median | 194 | 0.532057104 | 0.316279041 | 0.092522403 |
|  | IVW | 194 | 0.414507481 | 0.257737348 | 0.107779254 |
|  | Simple mode | 194 | 0.331025034 | 0.767119706 | 0.66657428 |
|  | Weighted mode | 194 | 0.331025034 | 0.617402317 | 0.592466935 |
| UACR on HF | MR Egger | 56 | 0.074332653 | 0.291333624 | 0.799579504 |
|  | Weighted median | 56 | 0.17764391 | 0.128804505 | 0.167840907 |
|  | IVW | 56 | 0.064823994 | 0.103390569 | 0.530671238 |
|  | Simple mode | 56 | 0.37650601 | 0.230659734 | 0.108329208 |
|  | Weighted mode | 56 | 0.261793869 | 0.173224912 | 0.136438114 |
| HF on CKD | MR Egger | 50 | 0.031356598 | 0.151583515 | 0.836994064 |
|  | Weighted median | 50 | 0.127394745 | 0.049518551 | 0.010091824 |
|  | IVW | 50 | 0.091220757 | 0.04473634 | 0.041442531 |
|  | Simple mode | 50 | 0.156507483 | 0.112376194 | 0.169994901 |
|  | Weighted mode | 50 | 0.180949896 | 0.093775162 | 0.059455159 |
| HF on BUN | MR Egger | 49 | -0.011081432 | 0.018418434 | 0.550299088 |
|  | Weighted median | 49 | 0.003911924 | 0.004795132 | 0.414607988 |
|  | IVW | 49 | 0.008734272 | 0.005548243 | 0.115431748 |
|  | Simple mode | 49 | 0.005947721 | 0.010181208 | 0.561830693 |
|  | Weighted mode | 49 | 0.004929276 | 0.00856453 | 0.567611051 |
| HF on EGFR | MR Egger | 50 | -0.007306315 | 0.008218854 | 0.378452707 |
|  | Weighted median | 50 | -0.000941544 | 0.001980552 | 0.634505641 |
|  | IVW | 50 | 0.001581777 | 0.002478594 | 0.523359812 |
|  | Simple mode | 50 | -0.000844352 | 0.004013109 | 0.834229221 |
|  | Weighted mode | 50 | -0.001788087 | 0.003445205 | 0.606091809 |
| HF on UACR | MR Egger | 49 | 0.009675303 | 0.03781268 | 0.79916427 |
|  | Weighted median | 49 | 0.004900665 | 0.010851809 | 0.651557997 |
|  | IVW | 49 | 0.015053023 | 0.011516989 | 0.19120331 |
|  | Simple mode | 49 | -0.002671143 | 0.022973969 | 0.907925031 |
|  | Weighted mode | 49 | -0.005331125 | 0.018332973 | 0.772461387 |
