# Supplementary material for: Causal relationship from heart failure to kidney function and CKD: A bidirectional two-sample mendelian randomization study
Source: PLoS One. 2023 Dec 11;18(12):e0295532. doi: 10.1371/journal.pone.0295532 (PMC10712866; doi:10.1371/journal.pone.0295532)
Supplement: S10 Table — (DOC) [file pone.0295532.s010.doc]

**S10 Table. MR estimates of the causal association between HF and Kidney Function and CKD(after MR-presso)**

| MR estimates | method | nsnp | beta | se | pval | OR(95% CI) |
| --- | --- | --- | --- | --- | --- | --- |
| BUN on HF | MR Egger | 65 | -0.010167336 | 0.477053516 | 0.98306351 | 0.99(0.39,2.52) |
|  | Weighted median | 65 | 0.099021964 | 0.224380947 | 0.658987269 | 1.1(0.71,1.71) |
|  | IVW | 65 | 0.150994115 | 0.186196662 | 0.417400763 | 1.16(0.81,1.68) |
|  | Simple median | 65 | 0.096385542 | 0.21841301 | 0.658996221 | 1.1(0.72,1.69) |
|  | Weighted mode | 65 | 0.118103514 | 0.363504595 | 0.746314627 | 1.13(0.55,2.29) |
| EGFR on HF | MR Egger | 191 | 0.418581912 | 0.588104787 | 0.477498949 | 1.87(0.54,6.42) |
|  | Weighted median | 191 | 0.532278725 | 0.315787336 | 0.091880443 | 1.7(0.92,3.16) |
|  | IVW | 191 | 0.452949699 | 0.240603646 | 0.059760642 | 1.51(0.91,2.51) |
|  | Simple mode | 191 | 0.326127171 | 0.792721691 | 0.681241576 | 1.39(0.31,6.26) |
|  | Weighted mode | 191 | 0.382327824 | 0.589829272 | 0.517637639 | 1.39(0.42,4.67) |
| UACR on HF | MR Egger | 55 | 0.102823488 | 0.277345326 | 0.712307626 | 1.11(0.64,1.91) |
|  | Weighted median | 55 | 0.177015033 | 0.124330835 | 0.154521167 | 1.19(0.94,1.52) |
|  | IVW | 55 | 0.029259539 | 0.099358228 | 0.76838708 | 1.03(0.85,1.25) |
|  | Simple mode | 55 | 0.375185476 | 0.245026008 | 0.131556125 | 1.46(0.9,2.35) |
|  | Weighted mode | 55 | 0.249863069 | 0.170618629 | 0.148867901 | 1.28(0.92,1.79) |
| HF on BUN | MR Egger | 45 | -0.001000807 | 0.01222433 | 0.935129928 | 1(0.98,1.02) |
|  | Weighted median | 45 | 0.004209535 | 0.005048904 | 0.404420611 | 1(0.99,1.01) |
|  | IVW | 45 | 0.005667758 | 0.003700557 | 0.125622202 | 1.01(1,1.01) |
|  | Simple mode | 45 | 0.00625202 | 0.009836567 | 0.528336009 | 1.01(0.99,1.03) |
|  | Weighted mode | 45 | 0.00554212 | 0.008266982 | 0.506111532 | 1.01(0.99,1.02) |
| HF on CKD | MR Egger | 49 | 0.083968738 | 0.144081017 | 0.562821623 | 1.09(0.82,1.44) |
|  | Weighted median | 49 | 0.132335267 | 0.050054877 | 0.00819802 | 1.14(1.03,1.26) |
|  | IVW | 49 | 0.110892441 | 0.042669942 | 0.009353879 | 1.12(1.03,1.21) |
|  | Simple mode | 49 | 0.156312365 | 0.106294443 | 0.147935475 | 1.17(0.95,1.44) |
|  | Weighted mode | 49 | 0.1807106 | 0.088835713 | 0.047476094 | 1.2(1.01,1.43) |
| HF on EGFR | MR Egger | 44 | -0.006345685 | 0.005268061 | 0.23511814 | 0.99(0.98,1) |
|  | Weighted median | 44 | -0.001014758 | 0.001900566 | 0.593393953 | 1(1,1) |
|  | IVW | 44 | -0.000527468 | 0.001603719 | 0.742229075 | 1(1,1) |
|  | Simple mode | 44 | -0.000684518 | 0.004278616 | 0.873641403 | 1(0.99,1.01) |
|  | Weighted mode | 44 | -0.001214957 | 0.003917712 | 0.757968619 | 1(0.99,1.01) |
| HF on UACR | MR Egger | 46 | 0.028128164 | 0.027329761 | 0.309003968 | 1.03(0.97,1.09) |
|  | Weighted median | 46 | 0.004902656 | 0.0107639 | 0.648769673 | 1(0.98,1.03) |
|  | IVW | 46 | 0.010880067 | 0.008546487 | 0.203001872 | 1.01(0.99,1.03) |
|  | Simple mode | 46 | -0.001781202 | 0.02448561 | 0.942331544 | 1(0.95,1.05) |
|  | Weighted mode | 46 | -0.004853938 | 0.020737041 | 0.815991617 | 1(0.96,1.04) |
