# Supplementary material for: Causal relationship from heart failure to kidney function and CKD: A bidirectional two-sample mendelian randomization study
Source: PLoS One. 2023 Dec 11;18(12):e0295532. doi: 10.1371/journal.pone.0295532 (PMC10712866; doi:10.1371/journal.pone.0295532)
Supplement: S11 Table — (DOC) [file pone.0295532.s011.doc]

**S11 Table. MR estimates of HF effects on CKD**

**(Hypertension and Diabetes mellitus were excluded)**

| MR estimates | method | nsnp | beta | se | pval | OR(95% CI) |
| --- | --- | --- | --- | --- | --- | --- |
| HF on CKD | MR Egger | 43 | 0.111790365 | 0.141818459 | 0.435076495 | 1.12(0.85,1.48) |
|  | Weighted median | 43 | 0.136379194 | 0.051235379 | 0.007772015 | 1.15(1.04,1.27) |
|  | IVW | 43 | 0.117970677 | 0.044638558 | 0.008222415 | 1.13(1.03,1.23) |
|  | Simple mode | 43 | 0.143686844 | 0.098558146 | 0.152308631 | 1.15(0.95,1.4) |
|  | Weighted mode | 43 | 0.170867801 | 0.081708078 | 0.042597896 | 1.19(1.01,1.39) |
